# How common are single gene mutations as a cause for lacunar stroke? A targeted gene panel study

**DOI:** 10.1212/WNL.0000000000008544

**Published:** 2019-11-26

**Authors:** Rhea Y.Y. Tan, Matthew Traylor, Karyn Megy, Daniel Duarte, Sri V.V. Deevi, Olga Shamardina, Rutendo P. Mapeta, NIHR BioResource: Rare Diseases Consortium, Willem H. Ouwehand, Stefan Gräf, Kate Downes, Hugh S. Markus

**Affiliations:** From the Stroke Research Group, Department of Clinical Neurosciences (R.Y.Y.T., M.T., H.S.M.), Department of Haematology (K.M., D.D., S.V.V.D., O.S., R.P.M., W.H.O., S.G., K.D.), and Division of Respiratory Medicine, Department of Medicine (S.G.), University of Cambridge; and NIHR BioResource: Rare Diseases (K.M., S.V.V.D., O.S., R.P.M., W.H.O., S.G., H.S.M.), Biomedical Campus, Cambridge, UK.

## Abstract

**Objectives:**

To determine the frequency of rare and pertinent disease-causing variants in small vessel disease (SVD)-associated genes (such as *NOTCH3*, *HTRA1*, *COL4A1*, *COL4A2*, *FOXC1*, *TREX1*, and *GLA*) in cerebral SVD, we performed targeted gene sequencing in 950 patients with younger-onset apparently sporadic SVD stroke using a targeted sequencing panel.

**Methods:**

We designed a high-throughput sequencing panel to identify variants in 15 genes (7 known SVD genes, 8 SVD-related disorder genes). The panel was used to screen a population of 950 patients with younger-onset (≤70 years) MRI-confirmed SVD stroke, recruited from stroke centers across the United Kingdom. Variants were filtered according to their frequency in control databases, predicted effect, presence in curated variant lists, and combined annotation dependent depletion scores. Whole genome sequencing and genotyping were performed on a subset of patients to provide a direct comparison of techniques. The frequency of known disease-causing and pertinent variants of uncertain significance was calculated.

**Results:**

We identified previously reported variants in 14 patients (8 cysteine-changing *NOTCH3* variants in 11 patients, 2 *HTRA1* variants in 2 patients, and 1 missense *COL4A1* variant in 1 patient). In addition, we identified 29 variants of uncertain significance in 32 patients.

**Conclusion:**

Rare monogenic variants account for about 1.5% of younger onset lacunar stroke. Most are cerebral autosomal dominant arteriopathy with subcortical infarcts and leukoencephalopathy variants, but the second most common gene affected is *HTRA1.* A high-throughput sequencing technology platform is an efficient, reliable method to screen for such mutations.

Cerebral small vessel disease (SVD) accounts for around 25% of strokes in the form of lacunar strokes and deep intracerebral hemorrhages (ICH),^1^ and is the primary pathology underlying vascular cognitive impairment.^2^ In the majority of cases, it is a sporadic disease of aging related to hypertension and subsequent arteriosclerosis, but a minority of cases are due to rare genetic variants.^3^ The most common inherited form of SVD is cerebral autosomal dominant arteriopathy with subcortical infarcts and leukoencephalopathy (CADASIL) due to *NOTCH3* variants.^4^ More recently, other genes have been reported to cause similar phenotypes, including *HTRA1*, *COL4A1*, *COL4A2*, *TREX1*, *GLA*, and *FOXC1.*^5^ However, the frequency of these variants in populations with presumed sporadic SVD is unknown.

Identification of disease-causing variants currently largely relies on Sanger sequencing of the gene of interest. Often for cost reasons this involves sequencing a subset of exons, such as in CADASIL, where exons 3 and 4 are most frequently affected, and therefore preferentially screened.^6^ As the spectrum of monogenic SVD expands, testing on a gene-by-gene basis is not cost- or time-effective. High-throughput sequencing (HTS) panels using next-generation sequencing technologies allow simultaneous testing in multiple genes underlying a single disease phenotype in a more cost-effective manner and are increasingly being used in clinical practice.

In this study, we developed a HTS panel comprising 15 genes linked to the SVD phenotype. We evaluated the platform for disease diagnosis and to determine the frequency of monogenic disease-causing variants in a well-defined population with MRI-confirmed younger-onset lacunar stroke. This study evaluates both known disease-causing mutations and novel, potentially disease-causing variants.

## Methods

### Platform design

The gene panel was developed to include 7 genes known to be causal of SVD (*NOTCH3*, *HTRA1*, *FOXC1*, *COL4A1*, *COL4A2*, *TREX1*, *GLA*) as well as 8 genes associated with disorders with SVD-related phenotypes. These include familial cerebral amyloid angiopathy (*APP*, *CST3*, *ITM2B*), familial hemiplegic migraine (*ATP1A2*, *CACNA1A*, *SCN1A*), and connective tissue disorders (*ABCC6*, *COL3A1*). These disorders share clinical manifestations with monogenic forms of SVD (for example, lacunar stroke, MRI white matter hyperintensities [WMH], dementia, migraine with aura, and encephalopathy) and could therefore present similarly. For each gene, the transcript on which to report variants was selected based on size, RefSeq information, and previously reported variants, and submitted to the Locus Reference Genomic database.^7^ The capture design has previously been described by Simeoni et al.^8^

### Study population

The study population consisted of patients from the UK DNA Lacunar Stroke Study.^9^ A total of 72 specialist centers across the United Kingdom recruited unrelated patients of European ancestry with MRI-confirmed lacunar stroke occurring at or before the age of 70. The study was approved by the Multi-Centre Research Ethics Committee for Scotland (04/MRE00/36) and informed consent was obtained from participants. Stored DNA was available for 950 patients, all of whom were included in this study.

Lacunar stroke was defined as a clinical lacunar syndrome, with an anatomically compatible lesion on MRI (subcortical infarct ≤15 mm in diameter). All patients underwent full stroke investigations including brain MRI, carotid artery imaging, and ECG. Echocardiography was performed when appropriate. Patients were excluded if the cause of stroke was not SVD, including stenosis >50% in the extracranial or intracranial vessels; previous carotid endarterectomy; cardioembolic source of stroke defined according to Trial of Org 10172 in Acute Stroke Treatment criteria^10^ as high or moderate probability; cortical infarct on MRI; subcortical infarct >15 mm in diameter, as these can be caused by embolic mechanisms (striatocapsular infarcts); and any other specific cause of stroke (e.g., lupus anticoagulant, vasculitis, dissection, known monogenic cause).

All MRI scans and clinical histories were reviewed centrally by one physician (H.S.M.). The presence and extent of WMH was graded on T2-weighted or fluid-attenuated inversion recovery (FLAIR) scans using the Fazekas scale: 0 = none, 1 = mild, 2 = early confluent, 3 = severe confluent, as previously described.^11^ Lacunar infarcts were identified as a high signal lesion on acute diffusion-weighted imaging performed within 3 weeks of acute stroke, or as a cavitated hypodense lesion on T1 or FLAIR sequences. Cerebral microbleeds were identified on gradient echo sequences. Family history was collected for first-degree relatives (table 1).

**Table 1:**
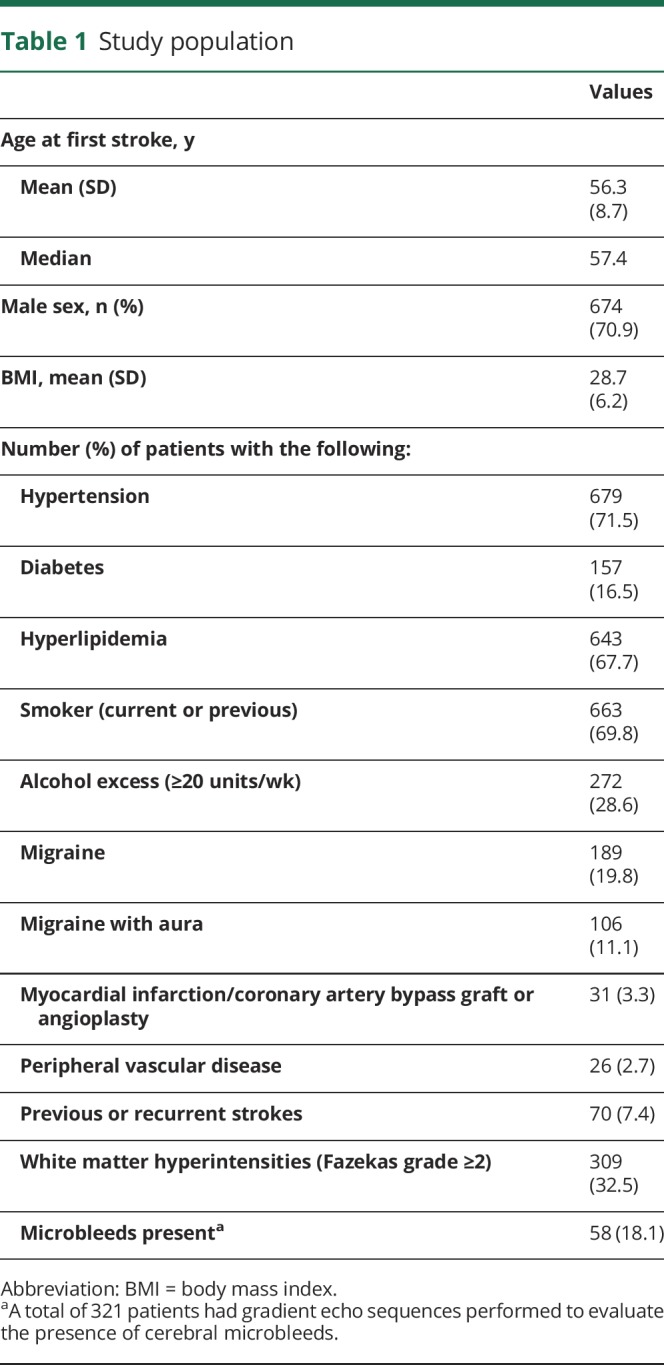
Study population

### Sample processing

DNA samples were processed as previously described.^8^

### Clinical bioinformatics

Sequence reads were processed as previously described.^8^ The filtering step was adapted to SVD: single nucleotide variants and indels were prioritized in the following: (1) minor allele frequencies in the genome aggregation database (gnomAD)^12^ <0.0001; (2) predicted impact according to SnpEff^15^ is high, moderate, or splice region; (3) presence in HGMD Pro (2017.2) or in curated locus-specific databases (in particular the Leiden Open-Source Variation Database [May 2017 version]^13^); (4) degree of deleteriousness according to Combined Annotation Dependent Depletion score^14^ ≥15. *NOTCH3* variants that resulted in the gain or loss of a cysteine residue in the EGF-like repeats were automatically prioritized, as they are known to cause CADASIL.^15^ The resulting variants were assessed according to the American College of Medical Genetics and Genomics guidelines^16^ and retained if classified as pathogenic, likely pathogenic, or of unknown significance.

Large copy number variants (CNVs) were called using a custom pipeline based on ExomeDepth 1.1.10^19^ as previously described.^17^

### Panel validation

The panel was assessed by comparing the results with those obtained by 2 independent methods: whole-genome sequencing (WGS) and *NOTCH3* and *GLA* sequencing.

Thirty-four samples sequenced using the panel were also sequenced using WGS, as part of the National Institute for Health Research (NIHR) BioResource–Rare Disease study. The NIHR BioResource projects were approved by Research Ethics Committees in the United Kingdom and appropriate national ethics authorities in non–United Kingdom enrollment centers. WGS was performed by Illumina (San Diego, CA) on HiSeqXTen generating 150 bp paired-end reads per lane with minimum coverage of 15X for at least 95% of the genome (30X on average). Reads were aligned to the GRCh37 build of the human genome reference using the Isaac Aligner, and variants were called using the Isaac VariantCaller.^18^ Variants in the 15 genes were analyzed following the same criteria as for the HTS panel.

Samples from all 950 patients had been previously screened for disease-causing *NOTCH3* and *GLA* variants.^9^ Exons in 3, 4, 5, 6, 11, 18, 19, and 22 of *NOTCH3* were screened using denaturing high-performance liquid chromatography (DHPLC), and in addition, exons 3 and 4 were screened using Sanger sequencing.^9^ Five patients with typical CADASIL-causing variants were identified. *GLA* was screened using high-resolution melt-curve analysis, covering all exons and intron/exon junctions, and one deep intronic region containing a known pathogenic variant. No Fabry-causing variants were identified.^9^

### Statistical analysis

Comparisons of variant frequency between patients with and without a family history of stroke and with and without confluent WMH were performed using the Fisher exact test. Analyses were performed using R statistical software (version 3.5.1).

### Data availability

Anonymized data will be made available upon reasonable request.

## Results

### Overall frequency of potentially disease-causing variants

Previously reported disease-causing variants and novel rare variants are shown in table 2.

**Table 2:**
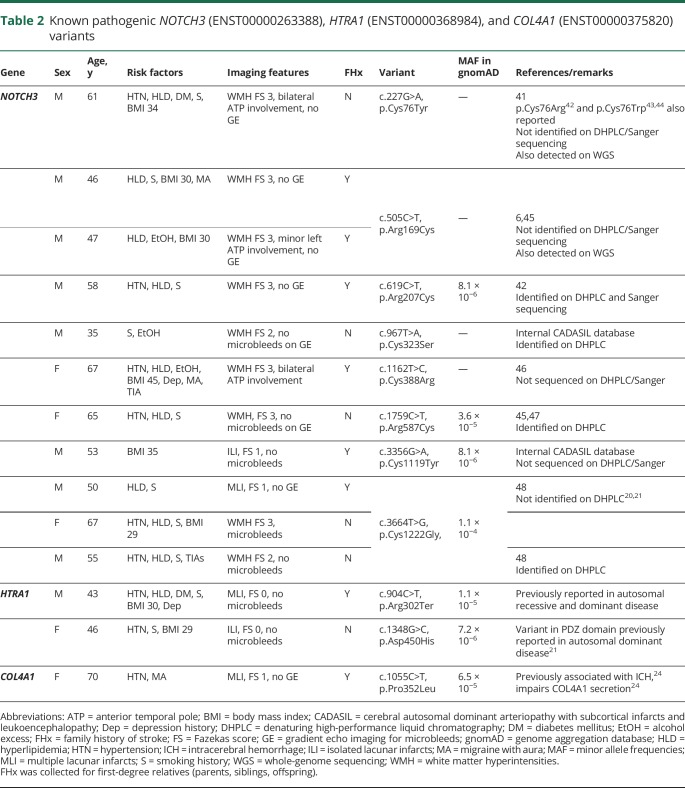
Known pathogenic *NOTCH3* (ENST00000263388), *HTRA1* (ENST00000368984), and *COL4A1* (ENST00000375820) variants

**Figure 1:**
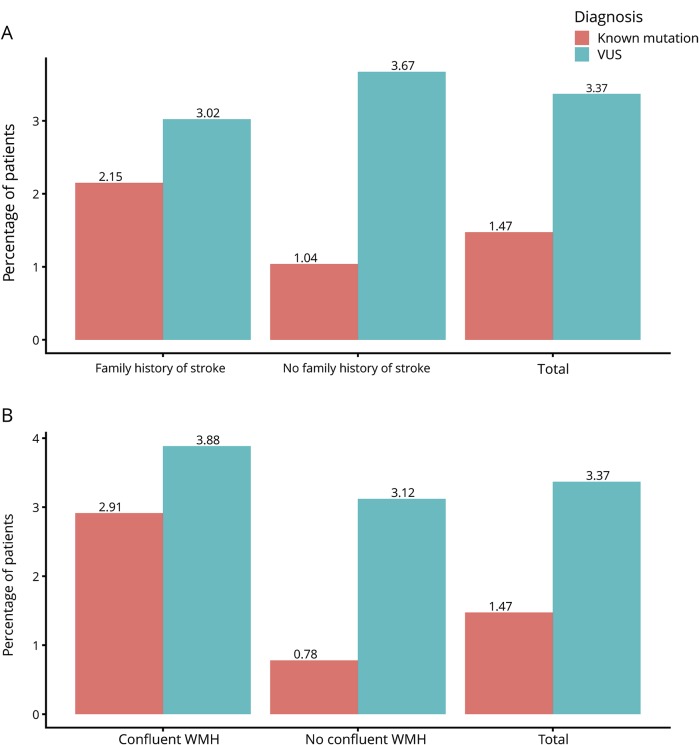
Family history of stroke and severity of white matter disease (A) Number (percentage of subjects tested) of known variants and variants of uncertain significance (VUS) identified in patients with and without a family history of stroke and (B) with and without confluent white matter hyperintensities (WMH) on MRI.

In the 7 known SVD genes, known disease-causing variants were identified in 14 individuals (1.5%); this represented 11 different mutations. The proportion of patients with a reported family history of stroke found to have mutations was higher (8 of 372 patients [2.2%]) than that in patients without a reported family history of stroke (6 of 578 patients [1.0%]), although this difference was not significant (*p* = 0.18) (figure 1A).

In addition, we identified 31 novel rare variants in the 7 known SVD genes. These were identified in 35 individuals. Excluding 2 *COL4A1* variants in 3 patients that were predicted to be benign in ClinVar (table 3), the overall frequency of novel variants was 3.4% (32 of 950 patients). There was no difference in the proportion of novel rare variants among patients with and without a family history of stroke (11 of 364 vs 21 of 572, respectively; *p* = 0.71) (figure 1A).

**Table 3:**
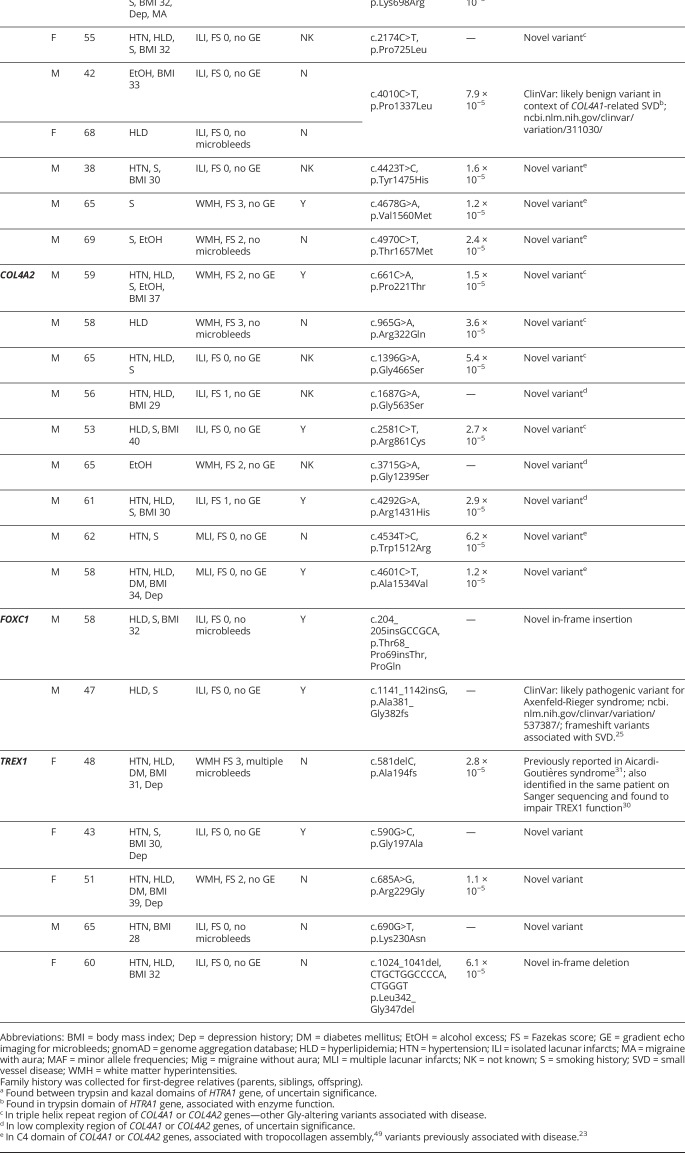
Rare, novel, or presumed benign variants identified in *HTRA1* (*ENST00000368984*), *COL4A1* (*ENST00000375820*), *COL4A2* (*ENST00000360467*), *FOXC1* (*ENST00000380874*), and *TREX1* (*ENST00000422277*) genes

To determine whether variants were more common in a particular phenotype of SVD, or in more severe cases, we examined variant frequency in those with confluent WMH. Of the 309 patients with confluent WMH on MRI (Fazekas score^10^ ≥2), 9 (2.9%) had a known disease-causing variant, compared with 5 of 641 (0.8%) in those without confluent WMH (*p* = 0.018). The proportions for rare novel variants of uncertain significance were 12 of 309 (3.9%) for those with WMH and 20 of 641 (3.1%) for those without (*p* = 0.57) (figure 1B).

### CADASIL

CADASIL is caused by cysteine-altering *NOTCH3* mutations in the epidermal growth factor–like repeat domains encoded by exons 2 to 24.^15^ Eight different cysteine-changing variants in exons 2–24 of *NOTCH3* were identified in 11 individuals (table 2). Previous *s*creening had identified 5 disease-causing variants,^9^ and these were again identified using this platform.

Of the 6 additional individuals with cysteine-changing *NOTCH3* variants, 2 variants in 2 individuals were previously missed by both DHPLC and Sanger sequencing.

The overall frequency of CADASIL-causing variants was 1.2% (95% confidence interval [CI] 0.6%–2.1%). Of patients with confluent WMH (Fazekas score ≥2) the frequency was 2.9% (9 of 309, 95% CI 1.5%–5.4%) compared to 0.3% of patients without confluent WMH (Fazekas score <2) (2 of 641, 95% CI 0.1%–1.1%) (*p* = 0.001).

Comparing age groups, the overall frequency of CADASIL-causing variants was 1.2% (95% CI 0.6%–2.5%) in patients ≤60 years and 1.1% (95% CI 0.4%–0.7%) in patients aged >60 years. Among patients with confluent WMH, the mutation frequency was 3.7% (95% CI 1.6%–8.3%) in patients ≤60 years and 2.3% (95% CI 0.9%–5.8%) in patients aged >60 years.

### Non-CADASIL monogenic small vessel arteriopathies

#### HTRA1

Missense and nonsense *HTRA1* variants have been reported in cerebral autosomal recessive arteriopathy with subcortical infarcts and leukoencephalopathy (CARASIL).^19^ Recently, heterozygous variants have also been identified in patients with an autosomal dominant form of SVD.^20,21^ Eight heterozygous missense variants and 1 nonsense *HTRA1* variant were identified in 12 individuals (1.3%, 95% CI 0.7%–2.2%). Two of these have previously been reported as disease-causing: p.Arg302Ter was reported in both recessive and dominant disease^19,20^; p.Asp450His was reported in autosomal dominant disease.^21^ p.Arg302Ter has been demonstrated to result in a mutant protein with 21%–50% of normal protease activity.^19^ There were no individuals with compound heterozygous or homozygous *HTRA1* variants.

Of the remaining novel *HTRA1* variants, 5 resided in the trypsin domain, with 1 (p.Arg227Trp) identified in 4 individuals. This variant is close to the trypsin active site in position 220. The clinical features of patients with *HTRA1* variants are provided in table 3.

Among patients with confluent WMH (Fazekas score ≥2), the frequency of rare *HTRA1* variants passing filters was 1.3% (4 of 309, 95% CI 0.5%–3.3%), similar to that in those without confluent WMH (1.2%, 8 of 641, 95% CI 0.6%–2.4%, nonsignificant difference). In younger patients (≤60 years), the frequency was 1.2% (7 of 574, 95% CI 0.6%–2.5%), and this value was similar in those older than 60 (5 of 376, 1.3%, 95% CI 0.5%–3.1%).

#### *COL4A1* and *COL4A2*

**Figure 2:**
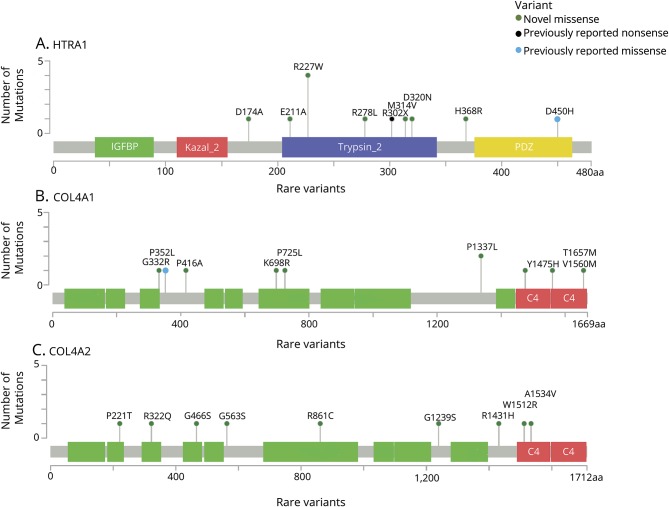
Rare variants identified in *HTRA1*, *COL4A1*, and *COL4A2* Rare variants identified in (A) *HTRA1* (ENST00000368984), (B) *COL4A1* (ENST00000375820), and (C) *COL4A2* (ENST00000360467).

*COL4A1* and *COL4A2* encode the α1 and α2 chains of collagen IV, respectively. Missense variants, typically but not always affecting the glycine residue in the repetitive Gly-X-Y regions, are associated with SVD and ischemic and hemorrhagic subcortical lacunar strokes.^22^ Variants affecting the C4 domain associated with tropocollagen assembly have also been associated with SVD.^23^ In 10 individuals (1.1%, 95% CI 0.6%–1.9%), we identified 9 missense *COL4A1* variants (figure 2B). There were no compound heterozygous individuals. One variant (p.Pro352Leu) was previously described in an individual with presumed sporadic ICH, and was also demonstrated to impair COL4A1 secretion into the extracellular space.^24^ Two other variants have been reported as likely benign in ClinVar (p.Gly332Arg and p.Pro1337Leu).

We identified 9 heterozygous missense *COL4A2* variants in 9 individuals (0.9%, 95% CI 0.5%–1.8%). None of these have previously been reported (figure 2C).

Variants identified in both *COL4A1* and *COL4A2* were found in various regions including the Gly-X-Y regions and the C4 domain associated with disease (table 3).

#### FOXC1

The *FOXC1* gene encodes the Forkhead box C1 transcription factor. Autosomal dominant missense, nonsense, and frameshift variants, as well as deletion or duplication of the locus (6p25), have been associated with ocular abnormalities described as the Axenfeld-Rieger syndrome.^25,26^ Some variants are also associated with white matter abnormalities.^25^ Two heterozygous predicted high-impact variants in *FOXC1* were identified in 2 individuals (0.2%, 95% CI 0.06%–0.8%): 1 novel in-frame insertion (p.Thr68_Pro69insThrProGln) and 1 frameshift variant (p.Ala381_Gly382fs) (table 3). The in-frame insertion was in a low-complexity region near the forkhead box domain, and has previously been reported in ClinVar as likely pathogenic for Axenfeld-Rieger syndrome (p.Ala381GlyfsTer147). Loss-of-function variants in *FOXC1* are known to be pathogenic and the frameshift variant is predicted to lead to premature stop codon 147 residues downstream.

#### TREX1

Retinal vasculopathy with cerebral leukodystrophy and systemic manifestations arises due to frameshift variants near the C-terminus of *TREX1*.^27^ Missense *TREX1* variants are also associated with Aicardi-Goutières syndrome,^28^ a form of pediatric-onset encephalopathy, and familial chilblain lupus.^29^ Three novel missense variants (p.Gly197Ala, p.Arg229Gly, p.Lys230Asn), 1 novel in-frame deletion, and 1 previously reported frameshift variant were found in 5 individuals (0.5%, 95% CI 0.2%–1.2%). The frameshift variant (p.Ala194fs) was also identified in the same patient in a separate study using Sanger sequencing (reported as p.Ala139Valfs*21 on a different isoform), and was found to nearly completely abolish TREX1 nuclease activity in vitro.^30^ This variant has been reported in a compound heterozygous case of Aicardi-Goutières syndrome in the DECIPHER database (patient 303873).^31^ (table 3).

#### Fabry disease: *GLA* variants

No pathogenic or likely pathogenic Fabry variants were identified.

### Copy number variants

Excluding CNVs with a Bayes factor lower than 20, only 1 CNV was detected in the 7 known cerebral SVD genes. This was a large duplication of both *COL4A1* and *COL4A2* in a single individual.

### SVD-related genes

Forty-five heterozygous variants in 8 genes associated with SVD-related disorders were identified in 47 individuals (table 4). Of note, 2 novel variants were identified in cystatin 3 (*CST3*), where variants are known to cause familial cerebral amyloid angiopathy, Icelandic type^32^: 1 stop-gained and 1 frameshift variant, both at position 93 (p.Glu93* and p.Glu93fs). Lacunar stroke is not an established manifestation of the disease, indicating that more investigations are needed to assign pathogenicity to these variants.

**Table 4:**
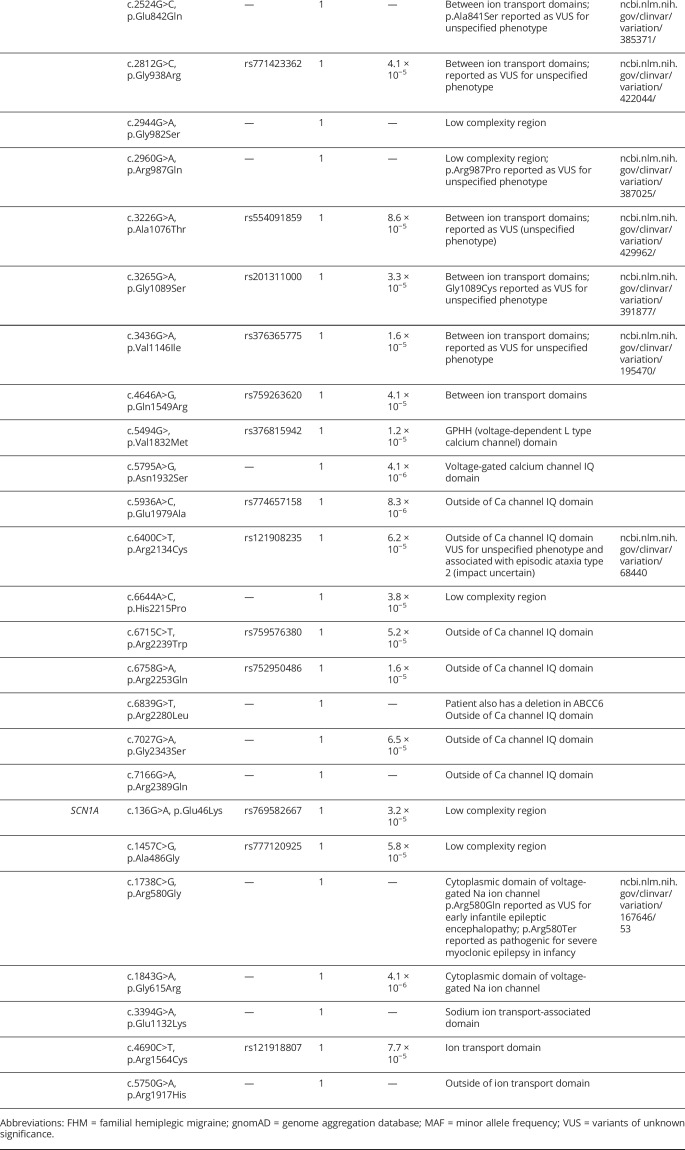
Rare variants identified in genes associated with phenotypes related to small vessel disease: *COL3A1* (ENST00000304636), *APP* (ENST00000346798), *CST3* (ENST00000376925), *ITM2B* (ENST00000378565), *ATP1A2* (ENST00000361216), and *CACNA1A* (ENST00000361216), *SCN1A* (ENST00000375405)

### Validation of panel

In the 34 individuals sequenced by WGS, 2 were found to harbor CADASIL-causing *NOTCH3* variants. These were also detected by the HTS platform (table 2). No other exonic variants passing the filters could be identified in the remaining 32 individuals, either by WGS or by targeted sequencing. In addition, 5 cysteine-changing *NOTCH3* variants had been detected on prior screening, all of which were also identified by the platform.

## Discussion

We developed an HTS panel for 7 genes known to be implicated in monogenic SVD and 8 genes associated with related phenotypes that can enter in the differential diagnosis of monogenic SVD. Validation against other techniques found the panel to be as sensitive, if not more sensitive than conventional screening methods, and detected all previously identified *NOTCH3* variants, including some missed on previous screening.

*NOTCH3* variants, which underlie CADASIL, the most common cause of monogenic SVD, were identified in about 1% of patients with apparently sporadic SVD aged 70 or under. The yield rose to 3.7% if analysis was limited to younger (age ≤60) patients who also had confluent WMH.

A number of other monogenic forms of SVD have been reported, and although they appear to be rarer than CADASIL, their frequency in cases of lacunar stroke is unknown. Our study provides data on their prevalence in cases of symptomatic lacunar stroke.

We found no homozygous CARASIL-causing variants, but up to 1.3% of cases had potentially disease-causing heterozygous missense and nonsense variants, suggesting that *HTRA1-*associated autosomal dominant SVD is the second most common cause of familial SVD. These findings are consistent with previous smaller studies in which about 5% of patients with *NOTCH3*-negative familial SVD carry missense and nonsense *HTRA1* variants.^21,33,34^

In addition, we identified several rare variants predicted to be damaging in *COL4A1*, *COL4A2*, and *FOXC1*. One individual had a *COL4A1* variant (c.1055C>T, p.Pro352Leu), which has been described in an individual with sporadic ICH.^24^ However, without functional support and evidence of segregation with disease, the pathogenicity of this variant remains uncertain.

We identified a frameshift *FOXC1* variant (c.1141_1142insG, p.Ala381_Gly382fs). Given that *FOXC1* has 1 exon, the transcript containing this predicted premature stop codon may escape nonsense-mediated decay. Functional studies are required to confirm pathogenicity of this high-impact variant.

Fabry disease has been suggested as an underreported cause of young stroke,^35^ and has been associated with MRI features of SVD,^36^ but we found no Fabry variants in this cohort. This is consistent with recent data suggesting that its importance as a cause of early-onset cryptogenic stroke may have been overestimated.^37,38^

Our results highlight a major challenge in the use of HTS panels in clinical practice; namely, determining whether a variant is pathogenic or benign. In CADASIL, variant interpretation is aided by the knowledge that all known disease-causing variants are cysteine-altering. Assigning pathogenicity of variants in genes such as *HTRA1*, however, is challenging. Variant interpretation guidelines highlight the use of allele frequency in large public databases. This presents particular challenges in late-onset diseases such as SVD. Databases such as gnomAD,^39^ which are used to infer population-level frequencies of potentially disease-causing variants, include individuals drawn from different populations predominantly of European ancestry, with variable age distributions. For individuals who are young at the point of recruitment, it is unknown whether they might develop SVD in the future. In addition, such databases include affected populations from diseases such as dementia, which might include patients with SVD due to misclassification. Determining whether variants segregate with disease in families is important but often family members are unavailable.

The use of targeted HTS panels presents several advantages. While WGS, whole-exome sequencing (WES), and HTS panels can all provide clinically acceptable coverage, the higher read depth of HTS panels allows for the detection of high-confidence CNVs. HTS panels also allow for savings in terms of cost and analysis time, compared to WGS and WES.

Our study has limitations. Our analyses did not include sequencing a cohort of MRI-phenotyped unaffected individuals. Previous studies have used age- and sex-matched controls unscreened for disease as comparison.^40^ However, the value of such controls is questionable. Much of SVD is subclinical prior to cerebrovascular events and ruling out subclinical disease in such controls is not possible.

This study was performed in patients of European ancestry. It would not be possible to extrapolate these results to populations of other ancestries as variant frequencies may vary significantly in different populations. Similar studies should be performed in other populations, particularly as population databases expand to better represent these ethnicities.

Our results demonstrate that monogenic mutations account for about 1.5% of SVD stroke. Furthermore, they demonstrate the utility of targeted HTS platforms in the diagnosis of monogenic forms of SVD. We showed they could detect CADASIL-causing variants, suggesting they could replace commonly used Sanger sequencing techniques, with the added benefit of screening for other genes, such as *HTRA1*, shown in this study to be the second most common cause of monogenic SVD.

## Glossary

CADASIL: cerebral autosomal dominant arteriopathy with subcortical infarcts and leukoencephalopathy
CARASIL: cerebral autosomal recessive arteriopathy with subcortical infarcts and leukoencephalopathy
CI: confidence interval
CNV: copy number variant
DHPLC: denaturing high-performance liquid chromatography
FLAIR: fluid-attenuated inversion recovery
gnomAD: genome aggregation database
HTS: high-throughput sequencing
ICH: intracerebral hemorrhage
NIHR: National Institute for Health Research
SVD: small vessel disease
WES: whole-exome sequencing
WGS: whole-genome sequencing
WMH: white matter hyperintensities
